# Laser Technology in Dentistry: From Clinical Applications to Future Innovations

**DOI:** 10.3390/dj12120420

**Published:** 2024-12-23

**Authors:** Liliana Sachelarie, Roxana Cristea, Ecaterina Burlui, Loredana Liliana Hurjui

**Affiliations:** 1 Department of Preclinical Discipline, Apollonia University, 700511 Iasi, Romania; 2 Faculty of Medicine and Pharmacy, University of Oradea, 410073 Oradea, Romania; roxicristea98@yahoo.com; 3 Department of Clinical Discipline, Apollonia University, 700511 Iasi, Romania; ecaterinaburlui@gmail.com; 4 Department of Morpho-Functional Sciences I, Discipline of Histology, “Grigore T. Popa” University of Medicine and Pharmacy, 700115 Iasi, Romania; loredana.hurjui@umfiasi.ro

**Keywords:** laser, dentistry, photobiomodulation (PBM)

## Abstract

This narrative review comprehensively synthesizes laser technology’s clinical applications, advantages, and limitations in modern dentistry. The review of 67 articles published between 2018 and 2023 highlights the latest advancements, including photobiomodulation (PBM) for enhanced tissue healing and inflammation control, alongside innovative uses in implantology, endodontics, and teeth whitening. The findings underscore the transformative potential of lasers in improving dental treatment precision and patient outcomes while addressing the barriers to their widespread adoption, such as costs and training needs. This review emphasizes the integration of laser technology into routine clinical practice and identifies pathways for future innovations in dentistry.

## 1. Introduction

This narrative review examines the many clinical uses of lasers in dentistry, comparing them with traditional approaches. The 1960s saw the development of the crucial technology known as the laser, which has revolutionized the medical field, particularly in dentistry. Theodore Maiman discovered the first laser used in medical applications in 1960, paving the path for utilizing focused light beams for medical uses [1]. Recent advancements in photobiomodulation (PBM) and implantology, endodontics, and teeth whitening highlight the significant potential of laser technology [2]. Ongoing research continues to enhance the capabilities of this technology, paving the way for future integrated and efficient applications.

Dental applications of laser technology became feasible in the 1990s owing to its high precision and ability to reduce patient discomfort [2].

In this narrative review, a keyword search in PubMed, Scopus, and Web of Science databases identified 400 initial articles. This search was designed to collect a broad range of studies relevant to discussing laser technology applications in dentistry without following a specific systematic review protocol, thus reflecting a qualitative and inclusive approach to literature selection. Titles and abstracts were assessed for relevance by focusing on their clinical applications and innovations and the challenges of laser technology. Following this screening, 67 articles were classified as high relevance and therefore included in this review, which adds a qualitative synthesis of knowledge to date.

We structured the literature selection process using a PICO question: “How do laser technologies influence dental treatments (Intervention) in patients (Population) compared to traditional methods (Comparison), and what are the clinical outcomes obtained (Outcomes)? This approach allowed us to filter and select articles from the PubMed, Scopus, and Web of Science databases, focusing on the most significant and current research in the field of laser technologies in dentistry.

To collect relevant data from the literature, we searched PubMed, Scopus, and Web of Science databases using a series of specific keywords and combinations. In PubMed, we used the combinations “laser technology” and “dental treatments”; in Scopus, the keywords “dental laser” and “PBM” (photobiomodulation) combined with “laser dentistry”; and in Web of Science, we searched “laser applications” together with “dentistry” and “clinical outcomes”. Table 1 presents the keywords and combinations for identifying the relevant literature in the selected databases. This strategy aimed to cover the study area thoroughly and make it easy for other research teams to replicate the search.

The detailed description of the search method ensures that interested researchers can replicate the process, enabling independent verification and further exploration of our study’s findings.

The selection criteria included articles screened for relevance based on their focus on clinical applications, innovations, and challenges of laser technology in dentistry. Articles published between 2018 and 2023 focusing on clinical applications, technological advancements, or innovations in laser-assisted treatments were included. Studies unrelated to dental lasers, those with low methodological quality, and conference abstracts or opinion pieces were excluded.

After applying these criteria, 58 articles were included in this review, providing a qualitative synthesis of findings relevant to the advancements and challenges in laser technology in dentistry.

The application of the laser in dentistry became possible only in the 1990s when the benefits of its use in dental treatments were discovered, especially due to its high precision and reduced patient discomfort [2]. The principle of operation consists of generating monochromatic and coherent light, which is absorbed by various types of dental tissues such as enamel, dentin, and soft tissues [3]. As a function of laser type and wavelength, the energy can be used to cut, vaporize, or coagulate tissue. The most used lasers in dentistry are the carbon dioxide (CO_2_) laser, the erbium-doped yttrium aluminum garnet laser, and the diode laser [3,4]. The Er laser is one of the most widely used lasers in dentistry due to its effectiveness in treating hard and soft tissues [5].

The characteristics vary depending on the dental procedures [3,4,5]. The primary applications of lasers in dentistry have been periodontal treatments, which reduce inflammation and bleeding and promote rapid tissue healing [6]. Today, lasers are used in various procedures, including removing carious lesions, cavity preparation for fillings, root canal therapy, and tooth whitening [4]. Laser procedures are important because they are less invasive and often do not require local anesthesia, improving the patient experience [7].

Lasers treat hard and soft tissues precisely, minimizing collateral damage [6]. They also sterilize the work area during treatment, significantly reducing the risk of infections. However, one of the significant obstacles to the widespread adoption of laser technology in dentistry remains the high cost of the equipment and training required for its effective use [7].

Although lasers are currently a universal solution for all types of dental treatments and have brought many benefits to dentistry, in many cases, their use is complementary to traditional techniques, such as dental burs and other mechanical tools [8]. As laser technology continues to develop, its role in dentistry is anticipated to grow, providing more precise and effective solutions. Future advances could include improvements in performance, cost reduction, and greater integration of the laser into everyday treatments [4,8].

Lasers used in dentistry work by emitting a focused beam of light, which can interact with dental and gingival tissues through various mechanisms, including photothermal and photochemical [2]. This energy beam is absorbed by specific tissue chromophores, allowing for decontamination, gingival remodeling, or cutting soft tissue with high precision while reducing postoperative bleeding and pain. These unique properties make the laser a versatile and effective technology suitable for various dental procedures, from periodontal treatments to implantology and teeth whitening [9].

## 2. Clinical Applications of the Laser in Dentistry

Laser technology has become essential in dentistry because it performs various dental treatments on soft and hard tissues, offering better control and superior precision than traditional techniques [2]. Laser use in modern dental treatments has been possible since the 1990s, when its clinical advantages, such as high precision and significant reduction in postoperative discomfort for patients, were discovered [1].

The laser allows precise tissue sections, thus minimizing damage to adjacent structures and promoting faster recovery with less inflammation and edema [2]. Also, the laser reduces the need for local anesthesia in specific procedures, offering increased comfort to patients through the absence of the vibrations and noise specific to traditional dental burs [3]. In minimally invasive soft and hard tissue surgery, this technology helps reduce intraoperative bleeding and postoperative discomfort, which improves the overall patient experience [4].

In addition to the immediate clinical advantages, dental lasers contribute to better visibility of the operative field by maintaining a dry and sterile environment during interventions, thus reducing the risk of postoperative infections [1]. These benefits position laser technology as a superior alternative to traditional techniques in many dental interventions, potentially improving efficiency and comfort in modern dental treatments. Table 2 shows the main clinical applications of the laser in dentistry.

## 3. Lasers in Soft Tissue Surgery

Lasers in soft tissue surgery have become more common because of their advantages over traditional surgical methods. Lasers in dental medicine can interact with soft tissues due to their specific wavelengths absorbed by water and hemoglobin. These properties make lasers ideal for cutting, vaporizing, and coagulating soft tissues, providing excellent bleeding control and minimizing postoperative discomfort [4,9]. The laser beam cuts, coagulates, and precisely ablates soft gingival tissues. Controlled energy minimizes bleeding by coagulating blood vessels, promoting faster healing. Unhealthy areas are removed by ablation, while precise cutting edges protect the surrounding tissues.

Lasers in soft tissue surgery can be used in procedures such as gingivectomies, frenectomies, removal of mucosal lesions, and gingival tissue remodeling. In the case of gingivectomy, the laser removes excess hypertrophied gingival tissue with minimal bleeding due to the capacity of immediate coagulation of the small blood vessels. Also, the procedure is more comfortable for the patient, requiring less local anesthesia and a shorter recovery time [10]. One of the important aspects of soft-tissue laser surgery is the fact that faster healing occurs. The laser seals nerve endings and blood vessels during cutting, significantly reducing postoperative discomfort and the risk of infections. In a head-to-head comparison between traditional and laser surgery, patients receiving laser treatments reported fewer complications and faster recoveries [10]. The advantages and disadvantages of laser use in soft dental tissues are given in Table 3.

## 4. Lasers in Hard Tissue Surgery in Dentistry

The use of lasers in dentistry has gained popularity in recent decades due to the many advantages, including pain reduction, precision, and reduced recovery time [9].

In hard tissue surgery involving bones and teeth, lasers are used for a wide range of procedures, such as dental caries, gingivectomies, bone lesions, and periodontal surgery (Table 4).

The use of lasers in treating dental hard tissues is a continuously developing technology and offers multiple advantages compared with conventional techniques [18,19].

Another benefit of the laser is maintaining a dry cavity, as lasers provide effective hemostasis during surgical procedures, allowing better visualization of the working field and more precise control [18]. The low risk of postoperative infections is another notable advantage due to the laser’s antimicrobial properties, which help decontaminate the treated area [20,21]. In addition to all this, patients enjoy a quick recovery, laser interventions being less invasive than conventional ones [22]

On the other hand, there are also some limitations. The high cost of laser equipment is an important factor to consider, as it can increase the cost of procedures for the patient [19]. In some cases, laser treatments can take longer than conventional ones, which is a disadvantage in terms of the longer time for certain procedures [21]. Also, specialized training is essential for correctly using the laser [18]. Another important limitation is the penetration depth, as the laser is not always ideal for accessing very deep areas of caries or removing certain types of dental restorations [20].

Laser technology brings numerous benefits to dental practice, but high equipment costs remain a significant barrier to its widespread adoption. The purchase of a dental laser, especially a high-performance one such as an erbium or carbon dioxide (CO_2_) laser, can cost between USD 10,000 and 80,000, depending on the type and functionality of the equipment [13]. This cost includes not only the initial price of the equipment but also the subsequent expenses for maintenance and calibration, which can become burdensome for small clinics or individual practices [13].

Another financial challenge is training staff to correctly and safely use dental lasers. Because laser technology requires specialized training to achieve maximum efficiency and minimize the risks of complications, dentists must participate in training or certification programs, which may incur additional costs and involve a considerable time investment [14]. Without this training, the effectiveness of laser-assisted treatments can be compromised, and the risks of adverse effects increase significantly [12].

In addition to the acquisition and training costs, using laser technology implies a higher price per treatment for patients, which may limit their access to these treatments. Studies show that patients often bear a higher cost for laser-assisted procedures, which reduces the demand for these treatments, especially in middle- and low-income regions [22].

Despite these financial challenges, advances in laser technology could help lower costs as equipment prices drop and more accessible training programs develop. Recent studies point out that as technology advances and becomes more accessible, the costs associated with the dental laser may decrease, thereby increasing accessibility for a broader range of practitioners and patients [4].

The lasers most used in hard tissue surgery are Er (erbium) and Er, Cr (erbium, chromium). They effectively remove dental caries and cavity preparation and provide precise and minimally invasive cutting [19].

Lasers operate at various wavelengths in dentistry, influencing the penetration depths and the treatments’ effectiveness in hard and soft tissues. For example, the Er laser operates at approximately 2940 nm, a wavelength strongly absorbed by water, making it ideal for hard and soft tissue treatments [3]. In contrast, the Nd laser, which operates at 1064 nm, penetrates deeper into tissues and effectively decontaminates subgingival tissues, having a high affinity for pigmentation and hemoglobin [4].

The red spectrum, covering approximately 600–700 nm, is used in photobiomodulation and has limited penetration, making it ideal for superficial treatments. In contrast, the near-infrared spectrum (700–1400 nm) allows deeper penetration and is suitable for applications in periodontics and endodontics, where greater tissue penetration is required [7,10]. Thus, this spectral differentiation helps practitioners to select the right type of laser for each dental procedure, providing optimized results.

Dosimetry plays a vital role in laser applications in dentistry, as device parameters directly impact tissue effects. Lasers with the same wavelength can yield different outcomes based on factors like beam spot size, output power, and energy level. For instance, smaller beam spots concentrate energy density, enhancing the ablation effect on tissues, which is crucial for precision and efficiency in modern dental procedures [19,20,21].

The laser is used in various clinical applications such as treating dental caries, providing an effective method of caries removal with minimal loss of healthy dental tissue [18,19,20,21]. It is also used to seal dental fissures, improving the retention of the sealant by properly preparing the tooth surfaces [20]. In endodontic treatments, the laser can help open and clean the tooth canals, making it easier to access and clean the root canals [22]. In addition, the laser can be used for teeth whitening, accelerating the whitening process when combined with chemical whitening agents [20].

Although lasers in dentistry have brought numerous advantages, some significant limitations and challenges must be considered when implementing this technology in clinical practice. Among these limitations are the high cost of equipment, the need for specialized training, and restrictions in the applicability of treatments, which prevent widespread adoption of the laser.

One of the biggest obstacles to implementing laser technology in dentistry is the high initial cost of the equipment [20,21,22,23]. Dental lasers, especially high-tech ones like the Er and CO_2_, can be significantly more expensive than traditional dental instruments. This includes both the purchase price of the laser itself and the maintenance and upgrade costs of the equipment, which can be substantial. For this reason, small or individual dental practices may find investment in laser equipment financially unreasonable, especially in the absence of a large patient volume to justify the frequent use of this technology [23].

Another limiting factor is the need for specialized training to use the laser effectively and safely. Using a laser in dentistry requires in-depth knowledge of the correct power settings, wavelength, and type of laser depending on the procedure and the tissue being treated (dental or gingival). Physicians must also know the potential risks of burning or tissue damage if the equipment is used incorrectly. This learning curve can impede many dentists, requiring additional time and investment to obtain certifications and develop the necessary experience [24].

Although lasers are effective for various dental procedures, they are not universal and cannot completely replace all traditional instruments. For example, in restorative dental treatments, the laser is not always ideal for removing deep cavities or hard restorative materials such as amalgam or ceramic crowns. In such cases, conventional cutters remain more efficient and faster. Also, the laser has limitations in penetration depth, making it difficult to treat very deep caries or tooth structures that require removing a large amount of tooth tissue [25].

In addition, the use of the laser in bone surgery or extensive periodontal treatment may be restricted due to the laser’s reduced ability to penetrate deep enough into bone or other hard structures. Although research is exploring the use of lasers in these areas, standardized protocols have not yet been established to guarantee consistent results in each case [26].

In some cases, laser treatments may take longer than traditional methods. For example, Coluzz et al. show that in cavity removal procedures or cavity preparation for fillings, the laser may require a longer time to remove affected tooth tissue than a dental bur. This can be frustrating for patients and limit clinical efficiency when performing complex or multiple procedures in a single session [27].

Mathew et al. also considers a thermal injury risk if the laser is misused, especially at high power. If the proper wavelength and power settings are not selected for the procedure, the temperature may rise too high, causing irreversible damage to the tooth or gum tissue. In long-term procedures, close monitoring is required to prevent overheating and minimize potential adverse effects [28].

Although laser technology offers many significant benefits in dentistry, such as increased precision and patient comfort, the high costs, specialized training required, and limitations in the applicability of the procedures continue to be major challenges. These barriers make the widespread use of lasers restrictive for many dental practices. However, as technology advances and costs decrease, lasers will likely become more accessible and common in modern dentistry.

The use of the laser in various specialties of dental medicine opens new horizons for innovative and effective treatments, as shown in Table 5.

Disinfection of the oral cavity using lasers has recently gained increasing attention due to its potential to improve oral hygiene, reduce bacterial loads, and reduce reliance on traditional chemical disinfectants [22].

Studies have shown that diode, Nd, and erbium lasers effectively eliminate oral pathogens, particularly those linked to periodontal disease and peri-implantitis. Lasers provide several advantages in oral disinfection, including precision, minimal patient discomfort, and reduced damage to surrounding tissues [29,30,31,32].

**Table 5:** Lasers in dental medicine.

Direction	Use	Bibliographies
Prophylaxis	Laser descaling	[30]
Disinfection of the oral cavity	Disinfection of the oral cavity	[22,31]
Periodontology	Treatment of periodontal disease	[32,33]
	Gingivectomy procedures	
Orthodontics	Facilitation of tooth movement	[34]
	Elimination of injuries caused by the device	
Implantology	Bone preparation	[35,36]
	Disinfection of the implant area	
Endodontics	Root canal treatment	[37]
Oral surgery	Tooth extraction	[38,39,40]
	Soft tissue surgery	
Teeth whitening	Soft tissue surgery	[39]

Recent studies have highlighted the effectiveness of diode lasers in reducing bacterial counts in periodontal pockets, improving outcomes when combined with scaling and root planing procedures [32]. Similarly, Nd lasers have been shown to effectively target the pigmented bacteria contributing to periodontal infections, while erbium lasers have demonstrated dual functionality in tissue ablation and microbial reduction [30]. These findings underline the potential of laser-assisted therapy in enhancing periodontal treatment.

Laser disinfection has also proven beneficial in cases where traditional methods are limited, such as treating peri-implantitis and in patients with dental implants. The evidence suggests that combining laser therapy with conventional treatments can result in better clinical outcomes for peri-implantitis, a common complication of dental implants [33]. Additionally, lasers have been investigated for their antimicrobial properties in root canal disinfection, providing an alternative to chemical irritants like sodium hypochlorite [31].

Beyond reducing the bacterial load, lasers are known for promoting wound healing and reducing inflammation, making them valuable for disinfection and post-surgical recovery. The photobiomodulation effects of lasers have been explored for their ability to enhance soft tissue healing following periodontal or surgical interventions, which has further expanded their role in oral healthcare [29].

However, despite their many advantages, the widespread adoption of laser technology in dental practices faces certain barriers. The cost of laser equipment and the need for specialized training remain significant challenges [23]. Furthermore, while in vitro studies have consistently shown the antimicrobial efficacy of lasers, additional clinical trials are needed to validate these findings in diverse patient populations and to establish standardized protocols for laser use in oral disinfection [31].

Nevertheless, research conducted after 2020 underscores the increasing importance of lasers in oral hygiene and dental treatment. As technological advancements continue, lasers are expected to play an even more significant role in the future of oral disinfection and the overall management of oral health [30].

The use of lasers in dentistry has evolved significantly in recent years, offering new approaches to treating oral conditions and surgical procedures. Among the most notable innovations are improved infection control, less invasive procedures, and beneficial effects on tissue healing, all of which have been explored in recent studies [32,33,34].

A major innovation is using lasers to decontaminate tooth surfaces and periodontal tissues. Laser diodes are now being used to reduce the bacterial load in periodontal and peri-implant treatments, having the ability to destroy bacteria more precisely and efficiently than traditional methods [35,36]. Studies show that laser diodes are effective in reducing bacteria in periodontal pockets, contributing to the success of scaling treatment and root smoothing [34].

Laser technology is also used to stimulate tissue regeneration and accelerate postoperative healing. Photobiomodulation, which uses lasers to stimulate cell metabolism and promote healing, is gaining popularity in dental treatments. This process reduces inflammation and accelerates tissue repair after oral or periodontal surgery [35].

Another area of research is using lasers in endodontic treatments, especially root canal disinfection. Compared with chemical disinfection methods such as sodium hypochlorite, lasers can penetrate deeper into the dentin and more effectively remove bacteria from the root canal, improving the success rate of endodontic treatments [36].

Regarding implantology, lasers treat peri-implantitis, a common complication of dental implants. A recent study demonstrated that using Nd lasers can significantly improve the management of this condition by reducing inflammation and effectively eliminating bacterial biofilm from the surface of implants [37].

Recent clinical studies confirm the effectiveness of laser therapy in reducing inflammation and periodontal pocket depth. The LANAP (Laser-Assisted Periodontal Therapy) procedure has proven superior to conventional methods in obtaining new periodontal attachments [38,39,40].

In addition, lasers are used for minimally invasive oral surgery, in which the technology allows for more precise sections and causes less bleeding and postoperative discomfort. For example, erbium lasers are frequently used for the excision of gingival tissue with better control over the depth of cuts and a reduced risk of complications [38].

These advances demonstrate that lasers improve efficiency and comfort in dentistry and open new perspectives in minimally invasive treatments and tissue regeneration.

The outlook for laser uses in dentistry beyond 2023 is promising, given the recent advances in laser technology and its increasing acceptance in dental treatments [39].

Lasers are valuable tools for achieving minimally invasive treatments with more precise tissue control and significantly reducing postoperative discomfort. In addition, lasers offer new possibilities in disinfecting root canals, managing peri-implantitis, and stimulating tissue regeneration [40,41].

An important aspect is the increasing use of photobiomodulation, which helps to reduce inflammation and accelerate the healing of soft tissues after surgery in dental treatments [42]. Photobiomodulation (PBM), also known as low-level laser therapy (LLLT), has gained significant traction in dentistry due to its potential to promote tissue healing, reduce pain, and accelerate recovery [43]. PBM uses low-level lasers or light-emitting diodes (LEDs) to stimulate cellular processes without generating significant heat or tissue damage [39,40,41,42,43,44].

PBM is particularly useful in managing postoperative recovery after extractions, periodontal treatments, and dental implant procedures in dental applications [45]. It helps reduce inflammation, accelerates wound healing, and provides analgesia [46]. For instance, studies show that PBM can improve the healing of gingival tissues and promote bone regeneration in implantology, enhancing overall treatment success [40] (Table 6).

PBM also treats recurrent aphthous stomatitis, herpes simplex lesions, and temporomandibular joint disorders (TMDs) [46]. Its ability to reduce pain and inflammation while promoting tissue regeneration is a valuable adjunct in many dental procedures [41,42]. Also, a study in Lasers in Medical Science from 2023 evaluated the effect of PBM on bone remodeling, highlighting the benefits of this therapy in postoperative healing [42].

In summary, PBM offers a non-invasive therapeutic approach that enhances traditional dental treatments, making it a growing tool for modern dental care [43].

Table 6 summarizes the diverse clinical applications of photobiomodulation (PBM) in minimally invasive dental treatments and highlights their benefits.

Table 7 reports on the applications and benefits of lasers in dentistry, including the types of lasers used, their effects, and technological integration in modern dental treatments.

American Dental Association (ADA) guidelines recognize the applications of lasers in surgical and non-surgical procedures for their effectiveness in reducing bleeding, discomfort, and the risk of postoperative infections [67]. Also, the American Academy of Periodontology (AAP) supports using lasers in periodontal treatments, considering that lasers can help eliminate bacteria and stimulate the healing of periodontal tissues, especially in cases of advanced periodontitis [49,68].

Future developments also focus on lasers’ portability and accessibility in dental offices [56]. Reducing the cost of laser equipment and accomplishing the broader training of specialists will make lasers a standard option in most dental treatments [60,61].

## 5. Conclusions

This review combines recent technological progress with practical implementation methods, establishing a thorough framework for understanding the growing influence of lasers in contemporary dental practice.

The laser is revolutionizing dentistry with its exceptional precision, minimally invasive interventions, and ability to stimulate rapid healing. The technology reduces pain and speeds recovery, providing superior results in deep disinfection and personalized treatments. With the integration of artificial intelligence and increasing accessibility, the laser is the future of dental treatments, redefining comfort and efficiency for patients and doctors.

## Figures and Tables

**Table 1:** Search strategy used in databases for the literature on laser technology in dentistry.

Database	Keywords	Combinations
PubMed	laser, dentistry	“laser technology” and “dental treatments” or “photobiomodulation” and “dentistry”
Scopus	dental laser, PBM	“dental laser applications” and “clinical outcomes” or “PBM” and “dental health”
Web of Science	laser therapy, dental procedures	“laser therapy” and “dental care” or “laser” and “dental procedures”

**Table 2:** Main clinical applications of laser in dental medicine.

Application	Description	Bibliography
Soft Tissue Surgery	Using lasers for soft tissue procedures like gingivectomy reduces inflammation and promotes rapid healing.	Romanos, G.E.; Nentwig, G.H. (1999) [4]
Hard Tissue Surgery	Lasers are used to remove caries and reshape bone; they are effective on hard tissues like enamel and dentin.	Parker, S. (2007) [3]
Root Canal Therapy	Sterilizing root canals with the laser to eliminate bacteria, improving endodontic treatment.	Pham et al. (2021) [5]
Teeth Whitening	Speeding up the whitening process by activating bleaching agents with the laser.	Walsh, L.J. (2003) [7]
Pain Management and Biostimulation	Low-level laser therapy (LLLT) reduces pain and stimulates tissue after procedures.	Goharkhay, K. et al. (1999) [6]
Periodontal Treatments	Laser periodontal treatment to reduce periodontal pockets and stimulate tissue regeneration.	Parker, S. (2007) [8]
Oral Lesions and Precancerous Conditions Treatment	Laser removal of oral lesions and treating precancerous conditions with minimal scarring.	Myers, T.D. (1991) [2]

**Table 3:** Advantages and disadvantages of laser use in soft dental tissues.

Aspect	Description	Clinical Relevance	Bibliography
Advantages	Reduced bleeding due to the laser’s ability to coagulate blood vessels during surgery, leading to less intraoperative and postoperative bleeding [1].	In gingivectomy, laser reduces bleeding and eliminates the need for sutures.	Asnaashari, M.; Zadsirjan, S., 2014 [11]
Advantages	Minimized damage to surrounding tissues due to the laser’s precision, resulting in faster recovery and reduced swelling [2].	In frenectomy, minimal damage allows for faster healing and less patient discomfort.	Abu-Ta’a, M.; Karameh, R., 2022 [12]
Advantages	Lower risk of infection as the laser sterilizes the area during the procedure, reducing bacterial contamination [1].	Laser use in excising lesions or treating periodontitis reduces the risk of secondary infection.	Asnaashari, M.; Zadsirjan, S., 2014 [11]
Disadvantages	The high cost of laser equipment makes it less accessible for smaller dental practices and increases treatment costs for patients [3].	Smaller clinics may not afford laser technology, impacting the availability of laser treatments.	Tzanakakis et al., 2021 [13]
Disadvantages	Limited penetration depth in soft tissue surgeries can restrict the laser’s effectiveness in certain procedures [4].	Procedures requiring deep-tissue work may still necessitate conventional methods.	Naidoo, S.; Mulder, R., 2015 [14]
Disadvantages	Requires specialized training for dental practitioners, which may limit its widespread use in some clinics [2].	Clinics with insufficient training may face challenges in adopting laser technology.	Abu-Ta’a, M.; Karameh, R., 2022 [12]

**Table 4:** Advantages and disadvantages of laser use in hard tissue surgery.

Advantages	Disadvantages
Minimally invasive technique leading to less pain and discomfort.	The high initial cost of laser equipment [15,16].
Reduced need for anesthesia in certain procedures.	Requires specialized training for proper usage [15].
Decreased bleeding due to laser coagulation effect.	Limited application in certain dental procedures [17].
Reduced risk of infection due to laser’s antimicrobial properties.	Possible thermal damage to surrounding tissues if not used correctly [16].
Improved precision in tissue removal compared with traditional methods.	Not every time is suitable for deep caries removal [15,17].
Faster recovery time for patients.	The procedure took longer in some cases than traditional methods [17].

**Table 6:** Applications of photobiomodulation (PBM) in minimally invasive dentistry.

Application	Description	Clinical Relevance and References
Postoperative Healing	PBM accelerates tissue healing and reduces inflammation after surgeries such as gingivectomy or frenectomy.	It improves recovery time and reduces postoperative complications [44,45].
Bone Remodeling	PBM promotes osteoblast activity and supports bone regeneration in implantology.	Enhances implant stability and bone integration [46].
Pain Management	PBM alleviates postoperative pain by modulating inflammatory responses and neural pathways.	Improves patient comfort and reduces reliance on analgesics [47,48,49].
Periodontal Therapy	PBM aids in reducing pocket depth and regenerating periodontal tissues during LANAP procedures.	Supports minimally invasive management of periodontitis [47].
Root Canal Disinfection	PBM complements laser-assisted canal sterilization by improving tissue healing around treated roots.	Increases success rates of endodontic treatments [48].
Aesthetic Applications	PBM minimizes sensitivity during teeth whitening and supports enamel repair.	Improves patient satisfaction in aesthetic dentistry [49,50,51].

**Table 7:** Lasers in modern dental treatments.

Aspect	Details
Types of Lasers	Er:YAG [45], Nd:YAG [44,45,46,47], Diode [50,52]
Applications	Periodontal surgery, implant treatments, management of peri-implantitis [45,53,54], minimally invasive surgery [51,54]
Benefits	Precision in treatment, reduced bleeding, minimal discomfort, and faster healing [55,56,57,58]
Effects	Decontamination, coagulation, stimulation of healing, reduction in bacterial biofilm [55,59,60,61,62]
Technological Integration	3D imaging, AI for personalized treatment [62,63,64,65,66]
